# 1843. Intravenous versus Partial Oral Antibiotic Therapy in the Treatment of Uncomplicated Bloodstream Infection due to *Streptococcus* species

**DOI:** 10.1093/ofid/ofac492.1472

**Published:** 2022-12-15

**Authors:** Lynn Broermann, Majdi N Al-Hasan, Majdi N Al-Hasan, Sarah Withers, Kristina L Benbow, Taylor Ramsey, Katelyn Ogren, Meghan McTavish, William Webster, Hana R Winders

**Affiliations:** University of South Carolina College of Pharmacy, Greenville, South Carolina; University of South Carolina School of Medicine, Columbia, South Carolina; University of South Carolina School of Medicine, Columbia, South Carolina; Prisma Health, Greenville, South Carolina; University of South Carolina College of Pharmacy, Greenville, South Carolina; University of South Carolina College of Pharmacy, Greenville, South Carolina; University of South Carolina, Mooresboro, North Carolina; University of South Carolina College of Pharmacy, Greenville, South Carolina; University of South Carolina School of Medicine, Columbia, South Carolina; Prisma Health Richland, Columbia, South Carolina

## Abstract

**Background:**

Effectiveness of oral antibiotics for transition of therapy in uncomplicated bloodstream infections (BSI) due to *Streptococcus* species remains unclear. This retrospective cohort study examines effectiveness of partial oral antibiotic regimens in patients with uncomplicated BSI due to *Streptococcus* species compared to standard intravenous (IV) only therapy.

**Methods:**

Adult patients with uncomplicated BSI due to *Streptococcus* species from April 2016 through June 2020 in 7 hospitals within Prisma Health in South Carolina were evaluated. Patients who died within 7 days of BSI were excluded to reduce the impact of survival bias. Multivariate Cox proportional hazards regression was used to examine time to treatment failure, defined as a composite of all-cause mortality and BSI recurrence within 90 days.

**Results:**

A total of 222 patients with *Streptococcus* species BSI were included in the analysis. Overall, the median age was 62 years, and 116 (52.3%) were men. Beta-hemolytic streptococci (87; 39.2%) were the most common bloodstream isolates, followed by *S. pneumoniae* (76; 34.2%) and viridans group streptococci (59; 26.6%). Among this cohort, 99 patients received only IV antibiotics, and the remaining 123 received partial oral therapy. The median duration of therapy in both groups was 14 days, and median duration of IV antibiotics prior to oral transition was 4 days. Most patients in the partial oral group were transitioned to either oral beta-lactams (62; 50.4%) or fluoroquinolones (47; 38.2%). Of the IV only group, 46 (46.5%) required outpatient IV antibiotics. Treatment failure rates were 12.0% and 4.4% in the IV only and partial oral therapy groups, respectively (p=0.04). After adjustments for age, chronic comorbidities, and initial response to therapy within the first 72-96 hours, there was no difference in the risk of treatment failure in the partial oral compared to IV only group (hazards ratio 0.55, 95% CI 0.19-1.64; p=0.28).

**Conclusion:**

Transitioning patients from IV to oral antibiotics may be a reasonable strategy in the management of uncomplicated BSI due to *Streptococcus* species. Partial oral therapy does not seem to have a higher treatment failure rate than standard IV only therapy and may spare many patients from outpatient IV antibiotics.

**Disclosures:**

**All Authors**: No reported disclosures.

